# Impact of gender on how intensive care medicine residents experience their medical studies and training and perceive their specialty: a national survey

**DOI:** 10.1016/j.aicoj.2026.100099

**Published:** 2026-06-08

**Authors:** Cécile Aubron, Cécile Plaud, Florian Reizine, Caroline Hauw-Berlemont, Charlotte Salmon Gandonnière, Laetitia Bodet-Contentin, Muriel Sarah Fartoukh, Mercedes Jourdain, Julien Le Marec, Fabienne Tamion, Nadia Aissaoui, Stephan Ehrmann, Florence Boissier, Olfa Hamzaoui, the FEMMIR Group for the French Intensive Care Society

**Affiliations:** a Médecine Intensive Réanimation, Centre Hospitalier Régional et Universitaire de Brest, Université de Brest, France; b ENSTA (Ecole Nationale Supérieure des Techniques Avancées), Université de Brest; c Service de Réanimation Polyvalente, CH de Vannes, France; d Médecine Intensive Réanimation, Hôpital Européen Georges Pompidou, AP-HP, Université de Paris, France; e Médecine Intensive Réanimation, INSERM CIC 1415, CRICS-TriGGERSep FCRIN Research Network, CHRU de Tours, methodS in Patient-centered Outcomes and Health ResEarch (SPHERE), INSERM UMR 1246, Université de Tours, France; f Médecine Intensive Réanimation, Hôpital Tenon, APHP, Sorbonne Université, Faculté de Médecine Sorbonne Université, Paris, France. Groupe de Recherche Clinique CARMAS, Université Paris-Est-Créteil (UPEC), Créteil, France; g Univ-Lille, CHU Lille, INSERM U 1190, Intensive Care Unit, Lille, France; h Médecine Intensive et Réanimation Infectieuse, Hôpital Bichat-Claude Bernard, AP-HP, Paris, France; i Médecine Intensive Réanimation, Hôpital Universitaire de Rouen, Rouen, INSERM U1096 EnVi,Université Normandie, UNIROUEN, France; j Cardiology, Hôpital Européen Georges Pompidou, APHP, Université Paris Cité, Paris, France; k Médecine Intensive Réanimation, INSERM CIC 1415, CRICS- TriGGERSep FCRIN Research Network, CHRU de Tours, and Centre d’étude des Pathologies Respiratoires, INSERM U1100, Université de Tours, France; l CHU de Poitiers, Médecine Intensive Réanimation, INSERM, Centre d’Investigation Clinique CIC 14-02 IS-ALIVE, Université de Poitiers, F-86000 Poitiers, France; m CHU Reims, Unité de Médecine Intensive et Réanimation Polyvalente, Université de Reims Champagne-Ardenne, UR 3801 PPF, F-51100 Reims, France

**Keywords:** Intensive care medicine, Gender, Diversity, Sexual harassment, Resident

## Abstract

**Background:**

Parity has been reached among French residents in the intensive care medicine (ICM) specialty; however, concerns about underrepresentation of women in leadership position and gender discriminations remain. We hypothesised that perception of gender inequity differs between female and male ICM residents and increases along the ICM training.

**Methods:**

This nationwide observational closed-survey investigated how ICM residents experienced their medical curriculum, how they perceived the ICM specialty, and the potential reasons for women to be underrepresented in leadership position.

**Results:**

Among 113 residents who responded to the survey, 63 (55.6%) were females, and 85 (75.2%) were beginners. Twenty-nine (25.7%) answered to be always or often self-confident, and this number was lower in female than in male residents (14.3% versus 40.8%, p = 0.003). Women had less often than men the feeling to keep up with the situation along their medical training (39.7% versus 70.8%, p = 0.004). Societal injunctions to prioritise family over professional responsibilities were more often considered as barriers to leadership position by women than by men (75.8% versus 51.1%, p = 0.015). Fully trained residents agreed more frequently than beginners with the 2 following reasons associated with gender gap in ICM leadership position: men being more ambitious (31.1% versus 9.4%, p = 0.024) and professional environment discriminating against women (64% versus 46.2%, p = 0.017). Experience of non-physical sexual harassment was very common in female residents, with 74.6% of them reporting to have been directly subjected to jokes of a sexual nature (versus 28.6% of men, p = 0.001) and 49.2% of them to have been victims of allegations with humiliating connotation (versus 22.4% of men, p = 0.007).

**Conclusions:**

Female ICM residents reported more often the feeling of not coping with their medical training, the lack of self-confidence, and non-physical sexual harassment than men. Reported awareness of a less supportive institutional environment to women academic career and of difference in assertiveness between men and women increased along advancement in ICM training suggesting room for interventions.

## Introduction

Although gender parity has been achieved among French Intensive Care Medicine (ICM) residents, concerns remain that the underrepresentation of women in key and leadership positions will persist [1]. In France, one fourth of intensivist were female in 2020, and less than 8% of them were full professor [2]. Reasons contributing to this gender gap include the lack of female mentors, the difficulties to face logistics of maternity and motherhood in a demanding work environment, sexual harassment and societal injunctions [2].

Awareness of women under representativity, poor diversity and concerns about wellbeing in female doctors and minorities in ICM has led to the creation of task forces and working groups for diversity and equality in ICM scientific societies [1,3,4]. Specific actions with pro-active measures to support female intensivists and improve women mentorship and assertiveness have been claimed and implemented when possible [1,3,5]. As a consequence, the male-to-female speaker ratio at the French Intensive Care Society (Société de Réanimation de Langue Française, SRLF) annual conference improved from 5:1 in 2019 to 3:1 in 2025 when considering ICM and non ICM speakers (data from SRLF-FEMMIR). However, gender disparity among physician speakers at critical care conferences remains a concern [6]. Although gender parity in professional setting is essential, it does not mean that equality has been achieved. In a large North American observational study, access to full professor position and chair was significantly lower for women than men even after adjustment for women representativity. This unbalance increased throughout the study period while there were more female students, strongly suggesting that an increase in the number of female students does not lead to a proportional increase in the number of women at responsibility position [7]. Lewiss et al. described a multifactorial phenomenon where academic women physicians become invisible in the mid-career stage with a shift in equity perception between trainees or early careers physicians and mid-career stage academic women physicians [8]. In 2017, the French medical residency system was restructured, establishing ICM as an independent primary specialty with a dedicated 5 years program. In 2019, the French Intensive Care Society created the working group FEMMIR (https://www.srlf.org/femmir) for diversity and equality in ICM. One of the first actions of this task force was to increase awareness of women under representativity and of women’s perceived professional and personal fulfilment in ICM [2]. To better investigate gender gap reasons in ICM, to identify levers for mid and long term changes, we conducted a survey based on the hypothesis that perceptions of gender inequity might differ between female and male ICM residents, and also might evolve between the beginning and the end of ICM training. This study specifically focused on medical students and ICM trainees as there is an historical persistent female under representativity in this specialty, including in leadership positions [9,10], and as perceptions of gender stereotypes and discrimination during training may influence specialty choice and career orientation before professional establishment. This study aimed to describe 1) first how ICM residents experienced their clerkship and ICM medical training, 2) secondly, the perception of gender stereotypes and gender gap in ICM leadership positions by ICM residents, and 3) finally the presence and extent of sexual harassment during clerkship and residency in this population.

## Methods

### Study design

This nationwide observational closed cross-sectional online survey was developed as thanks to a partnership between the FEMMIR (FEmmes Médecins en Médecine Intensive Réanimation) and the department of sociology of the engineer college ENSTA (Ecole Nationale Supérieure des Techniques avancées), University of Brest. The FEMMIR is a working group that has been created within the French Intensive Care Society (Société de Réanimation de Langue Française, SRLF) in 2019 to overall promote women in ICM. It consisted of 12 members at the time of the study, including one resident, one sociologist, and several intensivists (mainly women, but also men). The sociology department of the engineer college ENSTA has expertise in the field of gender discrimination and diversity especially in setting where women are underrepresented.

The study was approved by the ethics committee of the French Intensive Care Society (CE SRLF 21-101), in accordance with the principles outlined in the Declaration of Helsinki. The study was at the initiative of the investigators who defined the survey’s design, implementations and analysis completely independently to any funding organization. This cross-sectional survey was performed according to the consensus based-Checklist for Reporting of Survey Study (CROSS) [11].

### Questionnaire development

The questionnaire, that is provided in the supplementary file, was first developed by three investigators (CA, CP and OH) and secondly adjusted based on the comments of the other members of the FEMMIR. The questionnaire was tested before being sent to participants by female ICM consultants. Five domains were investigated (1) baseline participants characteristics, (2) how participants experienced the medical curriculum, (3) perception of the ICM specialty, (4) potential reasons for women under representativity, and (5) sexual harassment. The survey contained up to 34 questions. Data on sexual harassment, that was defined as any situations in which a person imposes sexual or gender-based comments or behaviour on another, referred to any events occurring during medical studies (clerkship or residency) regardless of the ICM specialty.

The survey was set up in Limesurvey Version 3.28 (www.limersurvey.org). The checklist for reporting results of internet E-surveys was followed [12].

Respondents to the survey were not able to review and change their answers. To prevent multiple entries from the same individual a unique participation link was sent to each participant/registration. The responses were blinded and the individual responding could not be identified. Questions were either Likert scales, multiple or single choices.

### Participants and recruitment

Participants were the French ICM residents at the beginning of their training (November 2021 and November 2022) and at the end of their training (November 2022 and November 2023) from all French medicine schools. Beginners have already completed 6 years of medical studies, including 3 years of clerkship, and passed an exam at the end of the 6th year. The choice of specialty and of the medicine school location are based on the student’s rank at this exam. French residency has been remodelled in 2017, establishing ICM as an independent primary specialty with a dedicated 5-year training program. ICM training includes 8 periods of 6 months (3 in ICM, 1 in anaesthesia, 3 in any medical specialties and one left at free choice) and 12 months of junior ICU fellow. Participants at the end of their training were residents who had completed this 5-year training and validated all the courses and passed their medical thesis.

Participants were contacted by email via the resident’s mailing list provided by the College of Medical Intensive Care’s (Collège des enseignants de Médecine Intensive et Réanimation, CeMIR) that coordinates national medical intensive care educational curriculum. Participants were invited to take part in the survey via email with a concise survey introduction explaining purposes, duration and anonymity of the survey; the FEMMIR email address was also provided in the email in case of any questions. Similar information and informed consent were also explained at the beginning of the online survey. Participants could opt out of participating in the survey as their participation was voluntary.

Early-stage residents (beginners) were first contacted in November 2021 or November 2022, depending on the year they began their residency, while fully trained residents were contacted after completing their program (November 2022 and November 2023). Reminders were sent out 2–4 weeks after the first email to residents who did not participate, with a maximum of 2 reminders. The survey was advertised at the welcome courses provided to the early-stage residents and through the ICM residents association (ANJMIR, Association National des Jeune MIR) social media.

Data were collected anonymously.

### Statistical analysis

This cross-sectional nationwide survey was exploratory in nature. All questionnaires with completed demographic data and at least one additional domain were included in the analysis. No a priori sample size calculation was performed, as the study aimed to include all eligible French ICM residents during the study period.

Missing data were not imputed, and analyses were conducted on available cases. The proportion of missing data was low and balanced between comparison groups. Given the exploratory nature of the study and the number of comparisons performed, no correction for multiple testing was applied. Participation and completion rates were reported. Medians and interquartile ranges (IQR) or absolute and relative frequencies (%) were reported when applicable. Comparisons of survey responses between female and male residents were performed using Chi-square tests for categorical variables and Mann-Whitney U tests for continuous variables. Comparisons between early and fully trained residents were performed using the same tests. A p-value less than 0.05 was considered statistically significant. To strengthen results interpretation, the items "strongly agree" and "agree" were merged into "agree", while "strongly disagree" and "disagree" were merged into "disagree". Data were analysed using R version 4.3.1.

## Results

**Fig. 1:**
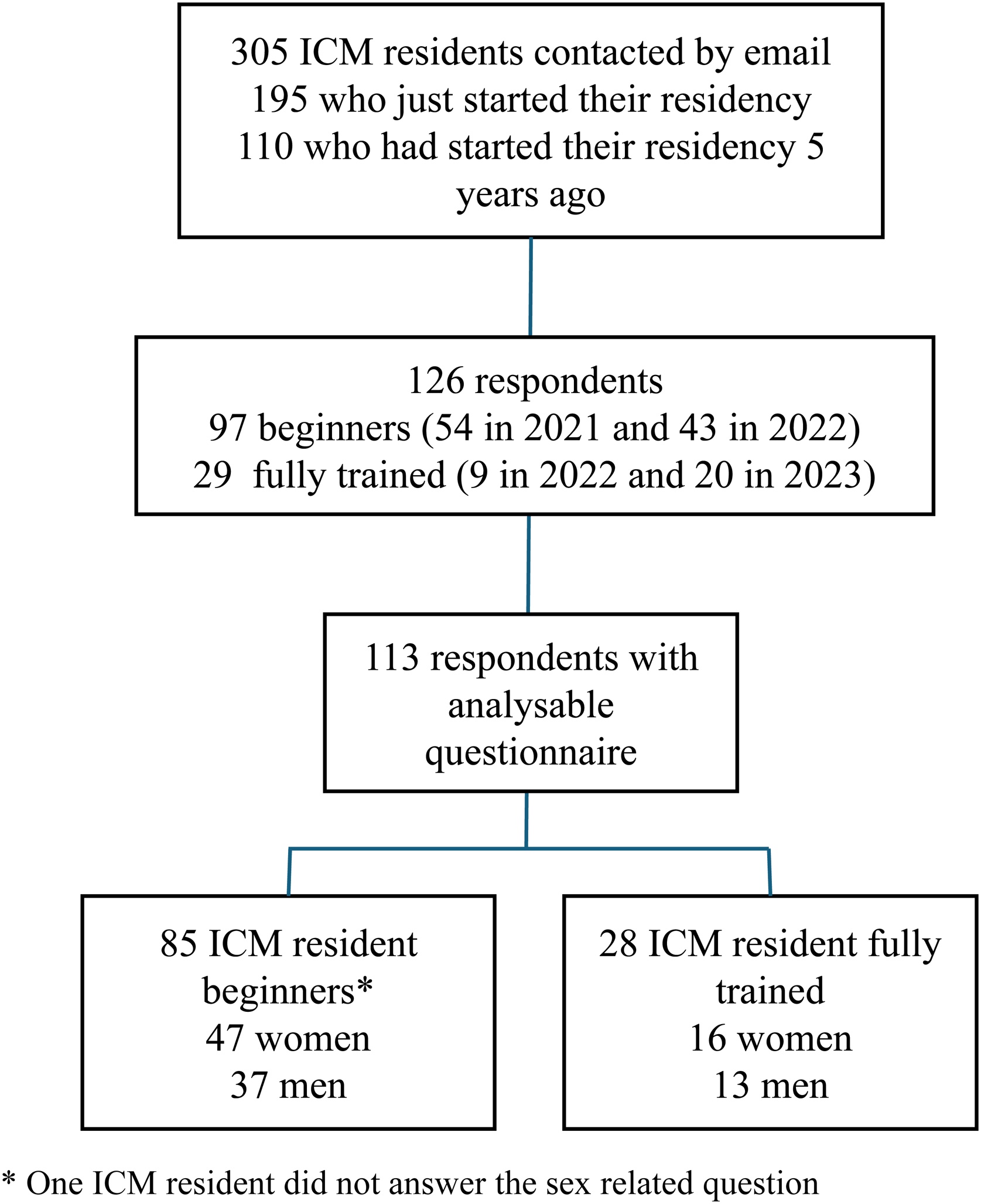
Study flow chart.

The questionnaire was sent to 305 ICM residents (195 early-stage residents (beginners) and 110 fully trained residents) (Fig. 1). 97 beginner residents (54 and 43 in 2021 and 2022, respectively) and 29 fully trained residents (9 in 2022 and 20 in 2023) answered demographics questions of the survey, but 113 responded to the questionnaire (85 beginners and 28 fully trained) leading to a participation rate of 43.6 % (85/195) for the beginner residents. Participation rate for fully trained doctors could not be determined as some residents temporarily interrupted their training for research or personal reasons. The 113 residents (beginners or fully trained) who responded to the questionnaire, answered more than 80% of the questions (Fig. 1).

The demographics characteristics of the whole study cohort is provided in supplemental Table S1. Among all respondents, 63 (56.3%) were women, and three-quarters were beginners. None of the respondents had children.

### ICM perception and determinants of choice for the ICM specialty

Most of the residents declared having chosen ICM based on their clerkship experience in ICU (85.8%) and/or on passion for the specialty (75.2%) without any difference between women and men (Table 1). Characteristics of ICM that were determinant for this specialty choice included the importance of clinical reasoning (92% of respondents), teamwork (86.7%) and the management of life-threatening emergencies (84.1%). The requirement for leadership abilities were mentioned by 37.2% respondents. Women declared significantly less frequently than men having chosen ICM for life threatening emergency management (74.6% of women versus 95.9% of men, p = 0.005) and for leadership abilities (28.6% of women versus 49% of men, p = 0.044) (Table 1).

**Table 1:** Determinants of choice and perception of the ICM specialty and comparison between women and men.

	All study cohort	Women	Men	
Variables	N = 113*	N = 63	N = 49	p
Choice of ICM was based on				
- Passion	85 (75.2)	46 (73)	38 (77.6)	0.741
- Advice of a teacher	17 (15)	10 (15.9)	7 (14.3)	1
- Family advice	4 (3.5)	2(3.2)	2 (4.1)	1
- Information meeting	15 (13.3)	9 (4.3)	5 (10.2)	0.719
- Medical student experience in ICU	97 (85.8)	54 (85.7)	42 (85.7)	1
Choice of ICM to treat life threatening emergency	95/113 (84.1)	47/63 (74.6)	47 (95.9)	0.005
Choice of ICM because leadership competence are required	42/113 (37.2)	18/63 (28.6)	24 (49)	0.044
Choice of ICM because of the high level of technicity	58 /113 (51.3)	28/63 (44.4)	30 (61.2)	0.116
Choice of ICM because of the importance of clinical reasoning	104/113 (92)	56/63 (88.9)	47 (95.9)	0.314
Choice of ICM because of the team work	98/113 (86.7)	53/63 (84.1)	44 (89.8)	0.552

* One respondent did not answer the question related to the sex.

The qualificative of ICM mentioned by more than 75% of respondents included spirit of decision, empathy, multi-disciplinarity, polyvalence and technicity, without difference between males and females (Supplemental Table S2). More than half of respondents (57.2%) considered that ICM made possible the management of personal and professional life, and around a third of them were concerned by the range of the hours and the number of shifts at night and on week-ends, without any difference between women and men (Supplemental Table S2).

### How did residents experience their medical training?

Most of the students (91.2%) felt successful in their studies, overall fulfilled (70.8%), well integrated (72.6%) and well supervised (69.9%), without difference between women and men. Although only 25.7% felt always and often self-confident, this number dropped to 14.3% in female respondents and was significantly lower than in male respondents (40.8%, p = 0.003). In the same way, the percentage of female residents who had the feeling to keep up with the situation during their medical training was lower than in male residents (39.7% versus 70.8%, p = 0.004). The reasons for this feeling were mainly stress and tiredness and were not different between women and men (Table 2). One third of residents declared having thought in the past of stopping their studies (38.1% of females versus 24.5% of males, p = 0.185), mainly because of self-applied pressure (75%), the issue in reconciling career and personal life (52.8%) and the feeling of not being able to do it (63.9%). This latter feeling was significantly more frequent among female residents than males (79.2% [19/24] vs. 33.3% [4/12], p = 0.002) (Table 2).

**Table 2:** How residents experience their medical studies and ICM training, and comparison between women and men.

	All study cohort	Women	Men	
Variables	N = 113*	N = 63	N = 49	*p*
Feeling of success in their studies/education	103 (91.2)	55 (87.3)	47 (94.9)	0.21
Feeling self-confident				0.003
- Always & often	29/113 (25.7)	9/63 (14.3)	20/49 (40.8)	
- Sometimes	56/113 (49.6)	33/63 (52.4)	22/49 (44.9)	
- Rarely & never	28 /113 (24.8)	21/63 (33.3)	7/49 (14.3)	
Felling fulfilled				0.485
- Always & often	80/113 (70.8)	45/63 (71.4)	34/49 (69.4)	
- Sometimes	23/113 (20.4)	11/63 (17.5)	12/49 (24.5)	
- Rarely & never	10/113 (8.8)	7/63 (11.1)	3/49 (6.1)	
Feeling well integrated among other students				0.973
- Always & often	82/113 (72.6)	46/63 (73)	35/49 (71.4)	
- Sometimes	24/113 (21.2)	13/63 (20.6)	11/49 (22.4)	
- Rarely & never	7/113 (6.2)	4/63 (6.3)	3/49 (6.1)	
Feeling well supervised				0.149
- Always & often	79/113 (69.9)	49/63 (77.7)	30/49((61.2)	
- Sometimes	28/113 (24.8)	11/63 (17.5)	16/49 (32.7)	
- Rarely & never	6/113 (5.3)	3/63 (4.8)	3/49 (6.1)	
Feeling well coached				0.281
- Always & often	62/113 (54.9)	37/63 (58.7)	25/49 (51.0)	
- Sometimes	36/113 (31.9)	16/63 (25.4)	19/49 (38.8)	
- Rarely & never	15/113 (13.2)	10/63 (15.9)	5/49 (10.2)	
Feeling to keep up with the situation				0.004
- Always & often	59/112 (52.7)	25/63 (39.7)	34/48 (70.8)	
- Sometimes	38/112(33.9)	25/63 (39.7)	12/48 (25)	
- Rarely & never	15/112 (13.4)	13/63 (20.6)	2/48 (4.2)	
No response	1		1	
Feeling Tired				0.536
Always or often	82/113 (72.6)	48/63 (76.2)	33/49 (67.4)	
Sometimes	26/113 (23)	13/63 (20.6)	13/49 (26.5)	
Rarely	5 /113 (4.4)	2/63 (3.2)	3/49 (6.1)	
Feeling stressed				0.371
Always, or often	72/113 (63.7)	35 (55.6)	26 (53.1)	
Sometimes	38 (33.6)	23(36.5)	15 (30.6)	
Rarely	13 (11.5)	5 (7.9)	8 (16.3)	
Feeling Lonely				0.5
Always or often	25 (22.1)	11 (17.5)	13 (26.5)	
Sometimes	38 (33.7)	22 (34.9)	16 (32.7)	
Rarely or never	50 (44.2)	30 (47.6)	20 (40.8)	
Feeling depressed				0.936
Always, often	20 (17.7)	11(17.5)	9 (18.4)	
Sometimes	35 (31)	20 (31.7)	14 (28.6)	
Rarely or never	58 (51.3)	32 (50.8)	26 (53)	
Feeling overwhelmed				0.299
Always or often	42/113 (37.2)	22 (34.9)	19 (38.8)	
Sometimes	45 (39.8)	29 (46)	16 (32.7)	
Rarely or never	26 (23)	12 (19.1)	14 (28.6)	
Having thoughts of stopping their studies	36/113 (31.9)	24/63 (38.1)	12/49 (24.5)	0.185

Data are presented as absolute value (percentage).

* One respondent did not answer the question related to the sex.

### Gender stereotypes and understanding about gender gap in leadership positions in ICM

Most of respondents agreed with the fact that a lack of female leader models (81/110; 73.6%) and a lack of support in general in the society for women to get responsibility position (80/111; 72.1%) contributed in the gender gap in ICM leadership position without difference between women and men (Table 3). Two-thirds of respondents believed that societal expectations for women to prioritize family and children and women’s own concerns about balancing professional and family life, represent barriers to women accessing to leadership positions (Table 3). The observed differences between men and women about social pressure to prioritize family over high-responsibility roles (75.8% vs. 51.1%, p = 0.072) and worries about reconciling professional and personal life (70.9% vs. 53.2%, p = 0.102) hinder women’s access to leadership positions did not reached significance. Female residents considered 2 times more than male residents that women’s lack of self-confidence contributes to gender gap in ICM leadership positions, although this difference was not statistically different (32.3% versus 15.2%, p = 0.116).

**Table 3:** Comparison of the reasons why women are underrepresented in leadership position in ICM and comparison between women and men.

	All study cohort	Women	Men	
Variables	N = 113*	N = 63	N = 49	p
Women worried about difficulty to reconcile personal life and career				0.102
Agree	70 (63.4)	44/62 (70.9)	25/47 (53.2)	
Disagree	33 (30)	16/62 (25.8)	17/47 (36.2)	
Neutral	7 (6.6)	2/62 (3.3)	5/47 (10.6)	
System discriminates against women				0.958
Agree	52/102 (51)	30/57 (52.6)	22/44 (50)	
Disagree	24 /102(23.5)	13/57 (22.8)	11 /44 (25)	
Neutral	26/102 (25.5)	14/57 (24.6)	11 (25)	
Women lack of self confidence				0.116
Agree	27/109 (24.8)	20/62 (32.3)	7/46 (15.2)	
Disagree	59/109 54.1)	31/62 (50)	27/46 (58.7)	
neutral	23/109 (21.1)	11 (17.7)	12 (26.1)	
Lack of female models in ICM				0.301
Agree	81/110 (73.6)	49/63 (77.8)	31/46 (67.4)	
Disagree	16/110 (14.6)	9/63 (14.3)	7/46 (15.2)	
neutral	13/110 (11.8)	5/63 (7.9)	8/46 (17.4)	
Women are told to prioritise family and that is not compatible with responsibilities				0.015
Agree	72/110 (65.5)	47/62 (75.8)	24/47 (51.1)	
Disagree	29/110 (26.3)	10/62 (16.1)	19/47 (40.4)	
neutral	9/110 (8.2)	5/62 (8.1)	4/47 (8.5)	
Random				0.954
Agree	3/101 (2.9)	2/59 (3.4)	1/42 (2.4)	
Disagree	93/101 (92.1)	54/59 (91.5)	39/42 (92.8)	
neutral	5/101 (5)	3/59 (5.1)	2/42 (4.8)	
Too many men in ICM				0.14
Agree	25 /105 (23.8)	13/61 (21.3)	12 /44 (27.3)	
Disagree	59/105 (56.2)	39/61 (63.9)	20/44 (45.4)	
neutral	21/105 (20)	9/61 (14.8)	12/44 (27.3)	
Less support for women to get responsibilities in general				0.919
Agree	80 /111 (72.1)	46/63 (73)	33/47 (70.2)	
Disagree	26/111 (23.4)	14 (22.2)	12 (25.5)	
neutral	5 /111 (4.5)	3(4.8)	2 (4.3)	
Women have less physical aptitude than men				1
Disagree	99 (87.6)	55 (87.3)	43 (87.8)	
Agree	14 (12.4)	8 (12.7)	6 (12.2)	

**Fig. 2:**
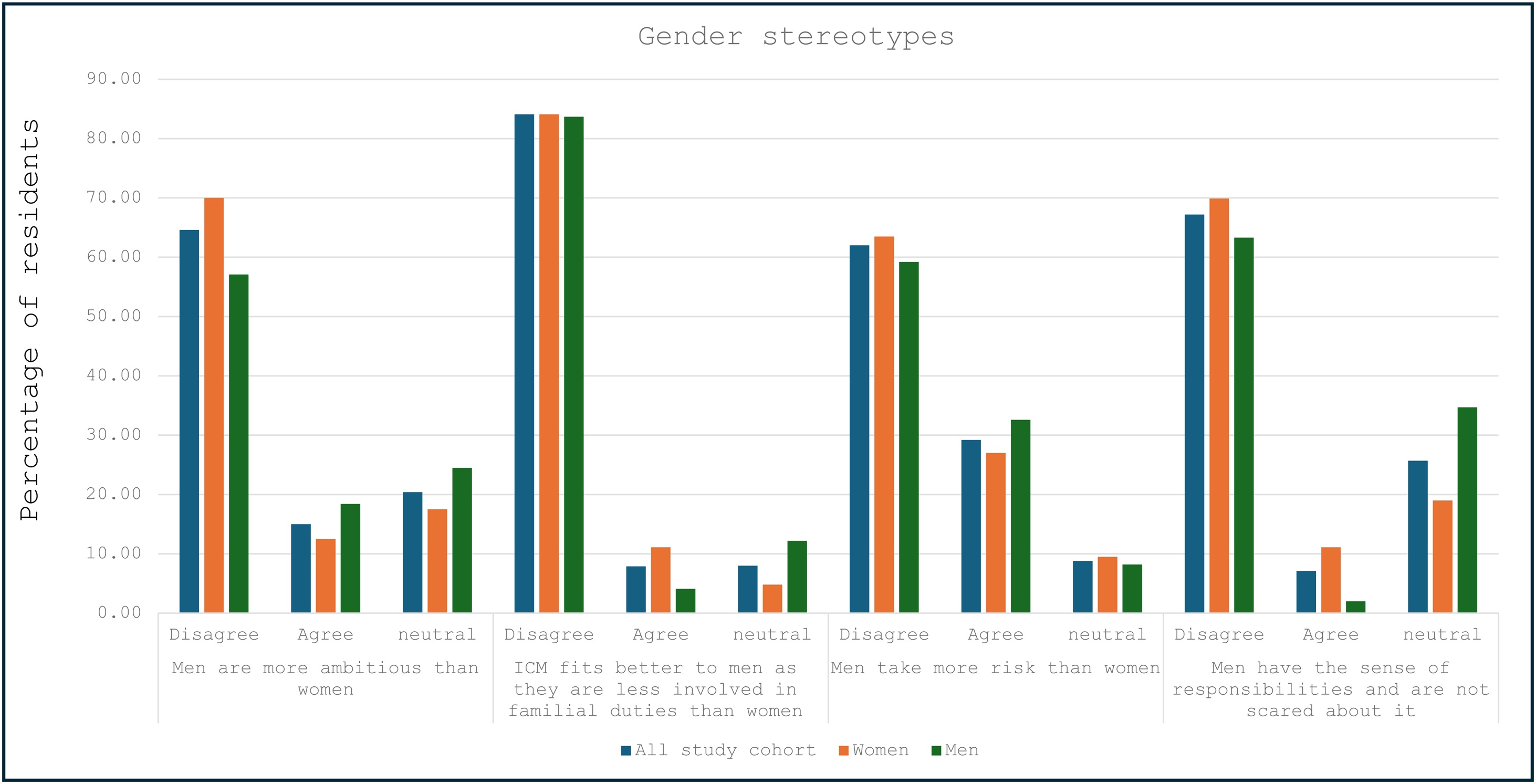
Gender stereotypes in residents and comparison between beginners and fully trained residents (p values were > 0.05 for all comparisons between beginners and fully trained residents).

When considering gender stereotypes in general, most of respondents disagreed with men fitting better with ICM because of being less involved in family duties (84.1%). They were around two third of residents to disagree with men being more ambitious (64.6%), having a stronger sense of responsibilities (67.6%). However, 29.2% of respondents believed that men take more risk in general than women. There was no difference between male and female participants in perception of gender stereotypes (Fig. 2).

### Sexual harassment

Women declared more frequently to have been victims of non-physical sexual harassment than men (Table 4), with 74.6% of them being directly subjected to jokes of a sexual nature (versus 28.6% of men, p = 0.001) and 49.2% of them victims of allegations with humiliating or degrading connotation (versus 22.4% of men, p = 0.007). In nearly half of the cases (47%), sexual harassment was reported to have occurred on multiple occasions, with a higher frequency among women than men (61% vs. 26.8%, p < 0.001) (Table 4). Although the perpetrators’ profile was highly variable, they were more frequently either a member of the medical team (95.3%), a teacher (57%) or a paramedic (48.6%) without differences between female and male residents. Overall, 38.6% of the respondents were shocked or hurt by sexual harassment, and most of them declared that were not enjoyable (81.2%), with significantly more women hurt and upset than male residents. Students who were victims of sexual harassment declared to confide themself first to friends and classmates (Table 4). One of the respondents was victim of exhibition and another of physical sexual harassment, both were women, and 35 (31%) respondents declared knowing someone having been victim of physical sexual harassment.

**Table 4:** Sexual harassment towards medical students or residents and comparison between women and men.

	All study cohort	Women	Men	
Variables	N = 113*	N = 63	N = 49	p
Jock of a sexual nature	61 (54.5)	47 (74.6)	14 (28.6)	<0.001
Insults of a sexual nature	7 (6.2)	6 (9.5)	1 (2)	0.219
Gesture of a sexual nature	16 (14.3)	10 (15.9)	6 (12.2)	0.785
Whistle with a sexual connotation	5 (4.5)	5 (7.9)	0	0.12
Messages of a sexual nature	9 (8)	8 (12.7)	1 (2)	0.088
Allegation with humiliation or degrading connotation	42 (37.5)	31 (49.2)	11 (22.4)	0.007
The respondent was not victim but knew someone else victim of harassment	41 (36.6)	13 (20.6)	28 (57.1)	<0.001
The respondent was not victim and did not know any victim of sexual harassment	12 (10.7)	3 (4.8)	9 (18.4)	0.045
How many times did sexual harassment happen?				
Never	38/100 (38)	13 (22)	25 (61)	
Once	15/100 (15)	10 (16.9)	5 (12.2)	<0.001
Several times	47/100(47)	36 (61)	11 (26.8)	
In what setting did sexual harassment happen?				
Hospital	62 (100)	46 (100)	16 (100)	NA
University campus	9 (14.5)	7 (15.2)	2 (12.5)	1
Student association	3 (6.4)	0	3 (21.4)	0.036
Students / Scholl year parties	7 (11.3)	6 (13)	1 (6.2)	0.779
Outside medical studies	21 (33.9)	16 (34.8)	5 (31.2)	1
Witness of allegations towards women?				
Always and often	55/110 (50)	43/63 (68.2)	12/47 (25.5)	
Sometimes	38 (34.5)	17 (27)	21 (44.7)	<0.001
Rarely	14 (12.7)	3 (4.8)	11 (23.4)	
Never	3 (2.7)	0	3 (6.4)	
Perpetrator of allegations towards women				
Teachers	61 (57)	29 (61.9)	22 (50)	0.305
Paramedic	52 (48.6)	28 (44.4)	24 (54.5)	0.405
Medical team	102 (95.3)	62 (98.4)	40 (90.9)	0.179
Medical students	27 (25.2)	16 (25.4)	11 (25)	1
Patients/family	38 (35.5)	23 (36.5)	15 (34.1)	0.959
Residents	45 (42.1)	29 (46)	16 (36.4)	0.425
Administration team	7 (6.5)	5 (7.9)	2 (4.5)	0.764
Feeling and thoughts about allegations towards women				
Shocking	18/101 (17.8)	8 (13.6)	10 (23.8)	
Not funny and hurting	21 (20.8)	17 (28.8)	4 (9.5)	
Not funny not hurting	43 (42.6)	28 (47.5)	15 (35.7)	0.004
Funny and cool	10 (9.9)	5 (8.5)	5 (11.9)	
No opinion or does not want to answer	9 (8.9)	1 (1.7)	8 (19)	
Who was a person resource				
Nobody	6 (5.4)	5 (7.9)	1 (2.0)	0.003
Admin Uni	1 (0.9)	1 (1.6)	0	0.59
Teacher	3 (2.7)	3 (4.8)	0	0.338
Classmate	28 (25)	23 (36.5)	5 (10.2)	0.003
Special committee	2 (1.8)	2 (3.2)	0	0.59
Family	14 (12.5)	12 (19)	2 (4.1)	0.037
Friend	25 (22.3)	21 (33.3)	4 (8.2)	0.003

### Difference between beginners and fully trained residents

Sex ratio was the same in beginners and fully trained residents (Supplemental Table S3). The determinants of choice of the ICM specialty were mostly similar between both groups, however beginner residents appeared to choose more often ICM because of a good clinical experience in ICU during their clerkship than fully trained residents, and we observed that they associated more often dynamism and polyvalence to the ICM specialty than fully trained residents (Supplemental Table S3).

We observed that beginners had less often the feeling of always or often keeping with the situation than fully trained residents (48.2% versus 64.3% p = 0.008); however, there was no other difference in their feeling about their medical training (Supplemental Table S4). Beginners associated less often ICM with wide ranges of hours at work than fully trained residents (22.6% versus 53.6%, p = 0.009) without any observed significant difference in the other parameters of work conditions perception, including the ability to reconcile personal and professional life (Supplemental Table S5).

We found that beginners agreed less frequently with the fact than men were more ambitious (9.4% versus 31.1%, p = 0.024) and took more risk than women (24.7% versus 42.9%, p = 0.055) (Fig. 2). In the same way, they think less often than fully trained residents that the gender gap in ICM leadership position is due to a system discriminating against women (46.2% versus 64%, p = 0.017) (Supplemental Table S6). There was no other significant difference in the understanding of the gender gap in leadership condition.

## Discussion

This national closed-survey reported that most respondents felt well supervised and well coached during their medical studies and training; however around two thirds of them felt always or often stressed. Female ICM residents reported more often the feeling of not coping with their medical training, the lack of self-confidence, and non-physical sexual harassment than men. Reported awareness of a less supportive institutional environment to women academic career and of difference in assertiveness between men and women increased along advancement in ICM training.

Our findings are aligned with those of a recent survey that reported 23% of low level of wellbeing among residents in all specialties [13]. Keeping with previous reports on science faculties, agentic traits, as ambitious and assertive, associated with ICM were more cited by men than women when they had to explain their ICM choice [14]. These differences, and those observed in the reasons given to explain the gap in ICM leadership position, might be the result of the socialization process by which women and men learn, very early in childhood, that children’s care and domestic tasks are a female duty, and that women have to anticipate the double carrier; whereas men have to invest primarily the professional area [15].

Our study highlights possible differences in self-perception between men and women. Assertiveness might be less developed in women than in men despite no statistical significance in the observed differences in the feeling of self-confident, possibly because of type 2 error. Feelings of “not being able” impact on career development as reported by De rosa et al. in a European survey mentioning that only half of ICM women accepted conference invitation [5]. Lower ability to self-advocate for procedural experience has been associated with female gender [16]. In the study by Olson et al., the reasons for unbalance in residents experience of procedural training were multifactorial, with possibly women being less encouraged by supervisors, and male gender associated with technicity [16]. Lack of self-confidence might also contribute to such differences.

Sexual harassment, hostile work place and subtle prejudices have been reported to be obstacles to career progression. In our study, female residents were more often victims of sexual harassment than men, with ¾ of women victims of jokes of sexual nature and half of them of allegation with humiliating or degrading connotation. These high rates are very closed to those reported previously [17]. Sexual harassment is well known to strongly contribute to self-depreciation, and to be an obstacle to career progression [5]. The ICM specialty as in other scientific professional fields therefore exposed to “the leaky pipe” phenomena [18]. The leaky pipe is a metaphor which illustrates the loss of women over the course of a scientific carrier. Sexual harassment as well as conciliation of private and professional carriers, or interiorisation of negatives female stereotypes (less leader, less competitive, more stressed and so on), explain that if parity is necessary to reach equality, it is not enough [18].

Our study highlights changes in gender perception between beginners and fully trained residents, with the latter considering men more ambitious and taking more risk, and considering more often the discriminative environment in general against women being responsible for gender gap in leadership position. These changes might illustrate the assimilation of medical values by students [19]. This is an important observation to acknowledge as women leave academic medicine more often, and at an earlier rank, than men [8,20,21]. Although this is likely to be multifactorial, some of the reasons might be accessible to interventions. Advancement in life with changes in preoccupations including a well balance family and professional career is a key element, but institutional environment that does not favour women career development, lack of models for combining career and family responsibilities and poor mentorship are accessible to interventions. Some research has highlighted the positive effect of the role model during a training program in science or on scholarly performances [22,23], or the likelihood of continuing coursework in sciences [24].

In our study, awareness of obstacles to women career seems well shared between male and female residents, that might be both the results of ICM feminization, but also a positive signal of changes in ICM perceptions, and possibly in the society in general.

### Future research and study implications

Further research is required to validate interventions to improve professional gender equality. For instance, research investigating institutional gender discriminations in payment, recruitment policies, maternity leave replacement that might participate to professional inequality should be conducted. In the same way, measures must be taken and evaluated to ensure women mentorship, especially at an early stage and midcareer, to minimise consequences of longstanding gender stereotypes and to enable women to deal with their personal life while having responsibilities, as recently promoted by an international panel of intensivists with interest in equity and equality [25]. Efforts must be pursued to banish sexual harassment [25]. Broader institutional and governmental policies are needed to ensure that maternity does not constitute a barrier to professional career development. Such measures should include access to hospital-based childcare facilities, dedicated breastfeeding rooms within ICU departments, transparent processes for recruitment and promotion, and official policies regarding work adaptations during pregnancy.

### Strengths and limitations

The study protocol followed key steps in its development and submission. It was submitted during 2 consecutive years increasing the sample size and its external validity. However, this study suffers limitations. As any survey, there is a participation bias. Response rate among fully trained residents could not be calculated as the list of fully trained students was uncertain (with some residents who had prolonged their residency, for, as examples, a year of master degree or pregnancy). The reasons for not participating were not recorded and the non-response error was not addressed. The study might be underpowered for some analyses especially those related to the comparison between beginners and fully trained residents. Indeed, only 28 fully trained residents were included, exposing to the risk of absence of significant difference in the comparisons between beginners and fully trained residents while a large sample size would have allowed to reach statistically significance level, especially for comparisons where there was a meaningful trend. The small sample size of this group questions also the representativity of the fully trained respondents. Some characteristics of sexual harassment were not available, they included specialties of the perpetuator (ICM or other specialties) and exact period (residency or clerkship) of occurrence. In the same way, the location where the residents lived (small versus large agglomeration) was not recorded. One participant chose the “no binary” proposition to the gender-related question, however our study did not investigate gender minorities. Additionally, comparisons between early-stage and fully trained residents were cross-sectional, and involved different cohorts rather than longitudinal follow-up of the same individuals. Therefore, observed differences may reflect cohort effects rather than true changes over time. Interpretation of the cross sectional comparison between beginners and fully trained residents should be approached with caution. Finally, multiple analyses exposed to the risk alpha inflation.

## Conclusions

In conclusion, in this nationwide observational closed-survey, most of residents agreed with the reasons for gender gap in leadership position, although women were more inclined to think that difficulties to reconcile personal life and career, injunction for prioritising family and children and the lack of female models were the sources of this gender gap. Non-physical sexual harassment remains ubiquitous and more frequent in female. The reported awareness of a hostile institutional environment to women academic career and of difference in assertiveness between men and women trend to increase along advancement in ICM training, suggesting room for interventions.

## Authors’ contribution

CA, CP and OH were involved in the study hypothesis generation. CA, CP, CHB, CSG, LBC, MSF, MJ, JLM, FT, NA and FB were involved in the conception of the study. FR performed the statistical analysis. CP, OH and CA wrote the first draft of the manuscript. All authors made substantial changes in the manuscript and approved the final version.

## Funding

This study was financially supported by La Cité du Genre-Université Paris Cité (ANR-18-IDEX-0001).

## Declaration of competing interest

The authors declare that they have no known competing financial interests or personal relationships that could have appeared to influence the work reported in this paper.
