# Supplementary material for: Impact of gender on how intensive care medicine residents experience their medical studies and training and perceive their specialty: a national survey
Source: Ann Intensive Care. 2026 Jun 8;16:100099. doi: 10.1016/j.aicoj.2026.100099 (PMC13284425; doi:10.1016/j.aicoj.2026.100099)
Supplement: Supplementary file 1 [file mmc1.docx]

**Supplementary file**

**Questionnaire for intensive care medicine residents**

**Q1** Where do you leave :

- With your parents or family members
- At your own place
- With your partner
- In share accommodation
- On University campus
- In Private student residence
- Other

**Q2** What socio-professional category does your parent 1 belong to ?

- Farmers
- Artisans, traders, business owners
- Managers and higher intellectual professions
- If parent 1 is a doctor, specify her/his position and speciality (enter text)
- Intermediate professions
- Employees
- Manual workers
- Retired persons
- Other persons not in employment

**Q3** What socio-professional category does your parent 2 belong to ?

- Farmers
- Artisans, traders, business owners
- Managers and higher intellectual professions
- If parent 2 is a doctor, specify her/his position and speciality (enter text)
- Intermediate professions
- Employees
- Manual workers
- Retired persons
- Other persons not in employment

**Q4** In general, do you consider yourself to be successful in your academic studies? (only one possible answer )

- Yes
- No

**Q5** In your opinion, what were the main factors that led you to choose the specialty of Intensive Care Medicine (ICM)? (multiple possible answers)

- I am passionate about intensive care
- I followed my teachers' advice
- I followed my family's advice
- I was convinced during information sessions on specialisations (e.g. career forums)
- I was convinced during my internship in a (medical) intensive care department.
- By default, I did not know what else to do
- I followed friends who wanted to do this medical specialty
- I don’t know, I don’t remember what motivated me
- I or someone close to me was hospitalised in ICM
- Others

**Q6** Among the following words, tick all those that you think best represent the ICM specialty.

- Patience
- Mental strength
- Decisiveness
- Courage
- Self-confidence
- Leadership
- Physical endurance
- Empathy
- Multidisciplinarity
- Technicity
- Adaptability
- Resilience
- Polyvalence
- Time availability
- Dynamism
- Physical strength

**Q7** On a scale of 1 to 6, how do you generally feel in you medical studies (1 never /2 rarely /3 sometimes/ 4 often/ 5 always/ 6 no opinion) (double-entry table)

- Confident
- Fulfilled
- Well integrated
- Well supervised
- Supported

**Q8** Would you say that you keep up to the task in your medical studies ?

- Never
- Rarely
- Sometimes
- Often
- Always
- without opinion

**Q9** If you replied one of the first four answers to the previous question (Q8), what are the main reasons ? (multiple choices possible)

- Financial issue
- Health issue
- Study conditions
- Familial issue
- My friendly and romantic relationships
- My professional future and integration
- Other
- I don’t know
- I don’t wish to answer

**Q10** On a scale of 1 to 6, how do you feel about your medical studies? (1 never/ 2 rarely/ 3 sometimes/ 4 often/ 5 always/ 6 no opinion)? (double-entry table)

- Tired
- Stressed
- Lonely
- Depressed
- Overwhelmed

**Q11** Since you started studying medicine, have you ever thought of stopping your training?

- Yes
- No
- I don’t wish to answer

**Q12** If you ever considered stopping your medical studies (after the first year), what were the reasons? (multiple choice possible)

- Financial issue
- Health issue
- The atmosphere within the promotion
- The relationship with the teaching staff or doctors
- A bad experience during my clerkship/studies
- Excessive workload
- A too intense pace of work
- The pressure that I put on myself
- The external pressure that I feel
- Lack of interest in these studies
- Difficulties balancing professional and personal life
- The stress related to patients management
- The feeling of not being capable
- I don’t know
- I don’t wish to answer

**Q13** In intensive care medicine, women are underrepresented particularly in key positions in various bodies (positions of collective responsibility in hospitals, universities or representative bodies for the discipline). In your opinion, how can this imbalance be explained? (multiple answers possible)

- Women don’t have the same physical abilities as men to succeed in intensive care
- They fear that the intensive care specialty will make it difficult to balance family and professional life.
- Early on in their lives, women are told that it is their responsibility to start a family and have children, and that this is not compatible with responsibilities.
- Women lack self-confidence and think they won’t succeed
- They lack female models in the intensive care medicine specialty
- The promotion system discriminates against women
- Women find that intensive care medicine is too male-dominated
- Girls are less encouraged (by school, family, society in general) to take on positions of responsibility.
- There is no justification, it is purely random

**Q14** You have choose intensive care medicine. Among the following items, tick those best illustrating why you did it (multiple answers possible).

- It is a specialty that deals with life threatening emergencies
- It is a specialty that requires leadership skills
- It is a highly technical specialty
- It is a specialty where clinical reasoning is essential
- It is a specialty where one works as part of a team
- Other

**Q15** Tick how much you agree or disagree with the following statements (used scale: completely disagree / rather disagree / neither agree nor disagree / rather agree / completely agree)

- Men are more ambitious than women
- The specialty of intensive care medicine is more suitable for men because they have less family responsibilities
- Men take more risks than women
- Men have the sense of responsibility and are not scared of it

**Q16** Tick how much you agree or disagree with the following statements (same scale as for Q15)

- In the intensive care medicine specialty, hour ranges are too long
- There are too many night shifts including week-ends
- In the intensive care medicine specialty, working conditions are difficult
- This profession is well regarded by society
- Intra-team conflicts could be frequent
- This profession is useful
- This profession allows to balance personal and professional life

**The next part of the survey is about sexual and gender-based harassment. This covers situations in which a person imposes sexual or gender-based comments or behaviour on another. This may include verbal or physical harassment.**

**Q17** Have you experienced gender-based or sexual verbal harassment during your studies, such as those listed below?

- Jock of a sexual nature directed towards you
- Insults of sexual a nature directed towards you
- Gesture of a sexual nature
- Whistling under the pretext of flirting
- Gender-based or sexual messages on the phone, the internet, social media or via sms/mms
- Any other allegation with a humiliating or degrading connotation directed towards you
- Never, but I know at least one student who has experienced this type of harassment
- Never, neither me nor anyone I know
- I don’t wish to answer

**Q18** How often, on average, do you/did you experience this type of verbal harassment in the course of your studies?

- Once
- Several times
- Never

**Q19** In what context did you experience this violence?

- During a hospital internship
- On the medical university campus
- In students associations
- At a promo evening, integration event/ welcome event…
- Outside the setting of my studies
- I don’t wish to answer

**Q20** Have you ever heard comments or jokes about women during you medical training ?

- Never
- Rarely
- Sometimes
- Often
- Always
- No opinion, or I don’t wish to answer

**Q21** If you have heard comments or jokes, who made them (multiple answers possible) ?

- Teaching staff
- Paramedical team
- Medical team
- Residents
- Medical students
- Patients and/or their families
- Administration

**Q22** If you answered rarely, sometimes, often or always to Q20, how did you react to these comments or jokes?

- Make me laugh, I find that they contribute to a lighter, more relaxed atmosphere
- Don’t make me laugh but they don’t hurt me, either
- Do not make me laugh and hurt me
- Shock me a lot.
- No opinion or I don’t wish to answer

**Q23** Did you ever experience physical sexual harassment during your medical training as listed below:

- Exhibition of a sexual nature
- Unwanted touching in private parts
- Not me but I know a friend who has been victim of this type of violence
- Not me and I don’t know anyone who has been subjected of this type of violence
- I don’t want to answer this question

**Q24** How many times do you/did you experience this type of **physical** harassment **in the course of your studies**?

- Never
- Once
- Several times

**Q25** If you have experienced physical harassment, who perpetrated it? (multiple answers possible)

- Teaching staff
- Paramedical team
- Medical team
- Residents
- Medical students
- Patients and/or their families
- Administration

**Q26** If you experienced sexual harassment (verbal or physical), have you talked about it ?

- I did not experience sexual harassment
- I have not discussed this with anyone
- I have discussed this with :

My university administration

A member of the teaching staff

My classmates

A faculty or hospital support unit

My family

A friend

Other

- I don’t wish to answer

**Q27** My personal situation

- Men
- Women
- Non-binary

**Q28** Enter your age (numeric entry)

**Q29** Are you  :

- alone
- as a couple

**Q30** Do you have one or more children ?

- Yes
- No

**Additional questions for fully trained residents**

**Q7 bis** How would you rate the quality of relationships during your ICM residency (on a scale of 1 to 5, very good/good/ bad / very bad/ no opinion)

- With female doctors
- With male doctors
- With female paramedic
- With male paramedic
- With male teacher
- With female teacher

**Q 8 bis** How would you rate the quality of relationships during your residency in medical ward (non ICM) (on a scale of 1 to 5, very good/good/ bad / very bad/ no opinion)

- With female doctors
- With male doctors
- With female paramedic
- With male paramedic
- With male teacher
- With female teacher

**Q11 bis** Since choosing your specialty (intensive care medicine), have you ever thought about changing it?

**Q12 bis** If you stopped your residency , what was the reason?

- Pregnancy
- Master
- Health
- Personal convenance
- Civil service
