# Supplementary material for: Impact of gender on how intensive care medicine residents experience their medical studies and training and perceive their specialty: a national survey
Source: Ann Intensive Care. 2026 Jun 8;16:100099. doi: 10.1016/j.aicoj.2026.100099 (PMC13284425; doi:10.1016/j.aicoj.2026.100099)
Supplement: Supplementary file 2 [file mmc2.docx]

Supplemental Tables

**Supplemental Table 1** Participants demographic characteristics and comparison between women and men

| Variables | Whole study cohort  N = 113* | **Women**  **N = 63** | **Men**  **N = 49** | **P** |
| --- | --- | --- | --- | --- |
| Age | 25 (24-28.5) | 25 (24-28) | 25 (24-29) | 0.368 |
| Beginner residents | 84/113 (74.3) | 47 (74.6) | 37 (75.5) | 1 |
| Socio-professional category of parent 1   - Farmers - Artisans, traders, business owners - Manager and higher intellectual profession - Employees - Jobless - Intermediate profession - Workers - At home   Parent 1 was a medical doctor | 2 (1.8)  2 (1.8)  79 (69.9)  12 (10.9)  3 (2.7)  9 (8)  3 (2.5)  3 (2.7)  20 (17.7) | 2 (3.2)  2 (3.2)  46 (73)  5 (7.9)  2 (3.2)  2 (3.2)  2 (3.2)  2 (3.2)  13 (20.6) | 0 (0)  0 (0)  33 (67.3)  7 (14.3)  1 (2)  6 (12)  1 (2)  1 (2)  7 (14.3) | 0.346 |
| Socio-professional category of parent 2   - Farmers - Artisans, traders, business owners - Manager and higher intellectual profession - Employees - Jobless - Intermediate - Workers - At home   Parent 2 was medical doctor | 0  7 (6.3)  50 (45)  22 (19.8)  2 (1.8)  22 (18.6)  0  8 (7.2)  9 (7.9) | 0  4 (6.3)  29 (46.0)  15 (23.8)  1 (1.6)  7 (11.1)  0  7 (11.1)  7 (11.1) | 0  3 (6.4)  21 (44.7)  7 (14.9)  1 (2.1)  14 (29.8)  0  1 (2.1)  2 (0.4) | 0.108 |

**Supplemental Table 2 ICM specialty perception and comparison between women and men**

| Variables | **All study cohort**  **N = 113*** | **Women**  **N = 63** | **Men**  **N = 49** | **p** |
| --- | --- | --- | --- | --- |
| What words fit the best with ICM  Patience  Mental strength  Decisiveness  Courage  Self-confidence  Leadership  Physical endurance  Empathy  Multidisciplinary  Technicity  Adaptability  Resilience  Polyvalence  Time Availability  Dynamism  Physical strength | 26 (23)  60 (53.1)  89 (78.8)  27 (23.9)  43 (38.1)  58 (51.3)  17 (15)  82 (72.6)  109 (92)  84 (74.3)  72 (63.7)  32 (28.3)  102 (90.3)  21 (18.6)  71 (62.8)  2 (1.8) | 13 (20.6)  35 (55.6)  47 (74.6)  16(25.4)  22 (39.4)  31 (49.2)  11 (17.5)  45 (71.4)  60 (95.2)  43 (68.3)  37 (58.7)  16 (25.4)  55 (87.3)  89 (14.3)  41 (65.1)  1 (1.6) | 13 (26.5)  24 (49)  41 (83.7)  10 (20.4)  20 (40.8)  27 (55.1)  6 (12.2)  36 (73.4)  43 (87.8)  40 (81.6)  35 (71.4)  16 (32.7)  46 (93.9)  12 (24.3)  29 (59.2)  1 (2) | 0.612  0.617  0.353  0.693  0.658  0.668  0.619  0.979  0.274  0.166  0.233  0.527  0.401  0.259  0.658  1 |
| Hours are too early and late  Agree  Disagree  Neutral | 34/112 (30.4)  49/112 (43.7)  29/112 (25.9) | 18/62 (29)  30/62 (48.4)  14/62(22.6) | 16/49 (32.6)  19/49 (38.8)  1449 (28.6) | 0.583 |
| To many night shifts and week ends  Agree  Disagree  Neutral | 36/110 (32.7)  34/110 (30.9)  40 /110(36.4) | 23/60 (38.3)  18/60 (30)  19/60 (31.7) | 13/49 (26.5)  16 (32.7)  20 (40.8) | 0.401 |
| Difficult work conditions  Agree  Disagree  Neutral | 45/112 (40.2)  40/112 (35.7)  27/112 (24.1) | 22/63 (34.9)  26 (41.3)  15 (23.8) | 23/48 (47.9)  14 (29.2)  11 (22.9) | 0.324 |
| Utile job  Agree  Disagree  Neutral | 109/111 (98.2)  1 (0.9)  1 (0.9) | 61/62 (98.4)  0  1 (1.6) | 47/48 (97.9)  1/48(0.1)  0 | 0.356 |
| Reconciling  personal and professional life  Agree  Disagree  Neutral | 63 /110 (57.2)  18 /110 (16.4)  29/110 (26.4) | 34 (55.7)  12 (19.7)  15 (24.6) | 29 (60.4)  6 (12.5)  13 (27.1) | 0.605 |

**Supplemental table 3** Comparison of reasons for ICM choice and ICM perception between fully trained and beginners residents

| Variables | All residents  N = 113 | Fully trained residents  N = 28 | Beginner residents  N = 85 | P value |
| --- | --- | --- | --- | --- |
| Age | 25 (24-28.5) | 30 (29-30.25) | 25 (24-25) | <0.01 |
| Female | 63 (56.2) | 16 (57.1) | 47 (56) | 1 |
| Successful feeling of their studies/education | 103 (91.2) | 25 (89.3) | 78 (91.8) | 0.986 |
| ICM choice   - Passion - Advice of a teacher - Family advice - Information meeting - Resident experience in ICU department - NOK hospitalised in ICU | 85 (75.2)  17 (15)  4 (3.5)  15 (13.3)  97 (85.8)  4 (3.5) | 17 (60.7)  6 (21.4)  2( 7.1)  2 (7.1)  20 (71.4)  2 (7.1) | 68 (80.0)  11 (12.9)  2 (2.4)  13(15.3)  77 (90.6)  4 (4.7) | 0.072  0.433  0.548  0.435  0.027  0.562 |
| What words fit with ICM  Patience  Mental strength  Decision maker  Courage, fearlessness  Self-confidence  Leadership  Physical endurance  Empathy  Multidisciplinary  Technicity  Adaptability  Resilience  Polyvalence  Time Availability  Dynamism  Physical strength | 26 (23)  60 (53.1)  89 (78.8)  27 (23.9)  43 (38.1)  58 (51.3)  17 (15)  82 (72.6)  109 (92)  84 (74.3)  72 (63.7)  32 (28.3)  102 (90.3)  21 (18.6)  71 (62.8)  2 (1.8) | 4 (14.3)  11 (39.3)  23 (82.1)  7 (25)  9 (32.1)  18 (64)  6 (21.4)  19 (67.9)  23 (82.1)  20 (71.1)  17 (60.7)  8 (28.6)  22 (78.6)  8 (28.6)  12 (42.6)  1 (3.6) | 22 (25.9)  49 (57.6)  66 (77.6)  20 (23.5)  34 (40)  40 (47.1)  11 (12.9)  63 (74.1)  81 (95.3)  64 (75.3)  55 (64.7)  24 (28.2)  80 (94.1)  13 (15.3)  59 (69.4)  1 (1.2) | 0.315  0.141  0.812  1  0.604  0.173  0.433  0.689  0.068  0.875  0.877  1  0.041  0.198  0.022  0.994 |

Others = default, follow a friend, NOK next of kin

**Supplemental Table 4** Comparison of how residents experienced their medical studies and training between beginner and fully trained residents

| Variables | All residents  N = 113 | Fully trained residents  N = 28 | Beginner residents  N = 85 | P value |
| --- | --- | --- | --- | --- |
| Self-confident  - always, often  - Sometimes  - Rarely, never | 29/113 (25.7)  56 (49.6)  28 /113 (24.8) | 12/28 (42.9)  10/28 (35.7)  6/28 (21.4) | 17/85(37.8)  46/85 (54.1)  22/85 (25.9) | 0.052 |
| Fulfilled  - always or often  - sometimes  - rarely or never | 80/113 (70.8)  23/113 (20.4)  10 (8.8) | 19/28 (67.9)  5 (17.9)  4 (14.2) | 61 (71.7))  18 (21.2)  6 (7.1) | 0.496 |
| Well integrated among students  -always or often  -sometimes  - rarely or never | 82 (72.6)  24 (21.2)  7 (6.2) | 21 (75)  4 (14.3)  3 (10.7) | 61 (71.8)  20 (23.5)  4 (4.7) | 0.349 |
| Well supervised  - always or often  - sometimes  - rarely | 79/113 (69.9)  28/113 (24.8)  6 (5.3) | 20 (71.4)  8 (28.6)  0 | 59 (69.4)  20 (23.5)  6 (7.1) | 0.332 |
| Well coached  - always or often  - sometimes,  - rarely or never | 62 (54.9)  36 (31.9)  51 (13.2) | 15 (53.5)  8 (28.6)  5 (17.9) | 47 (55.3)  28 (32.9)  10 (11.8) | 0.692 |
| Feeling to keep up with the situation  Always or often  Sometimes  Rarely or never  No response | 59 (52.2)  38 (33.6)  15 (13.2)  1 | 18/28 (64.3)  3 (10.7)  6 (21.4)  1 (3.6) | 41/85 (48.2)  35 (41.2)  9 (10.6)  0 | 0.008 |
| Feeling Tired  Always or often  Sometimes  Rarely | 82(72.6)  26 (23)  5 (4.4) | 24/28 (85.7)  3 (10.7)  1 (3.6) | 58/85 (68.2)  23/85 (27.1)  4/85 (4.7) | 0.183 |
| Feeling stressed  Always or often  Sometimes  Rarely or never | 72/113 (63.7)  38 (33.6)  13 (11.5) | 13/28 (46.4)  11 (39.3)  4 (14.3) | 49/85 (57.6)  27/85 (31.8)  9/85 (10.6) | 0.581 |
| Lonely  Always or often  Sometimes  Rarely or never | 25 (22.1)  38 (33.7)  50 (44.2) | 6/28 (21.4)  8/ 28 (28.6)  14/28 (50) | 19/85 (22.4)  30/85 (35.3)  36/85 (42.3) | 0.752 |
| Depressed  Always or often  Sometimes  Rarely or never | 20 (17.7)  35 (31)  58 (51.3) | 5/28 (17.9)  12/28 (42.9)  11/28 (39.2) | 15/85 (17.6)  23/85 (27.1)  47/85 (55.3) | 0.253 |
| Overwhelmed  Always, often  Sometimes  Rarely or never | 42/113 (37.2)  45 (39.8)  26 (23) | 10/28 (35.7)  10/28 (35.7)  8/28 (28.6) | 32 (37.6)  35 (41.2)  18/85 (21.2) | 0.712 |
| Thoughts of stopping the studies | 36 (31.9) | 10 (35.7) | 26 (30.6) | 0.786 |
| Reasons for stopping the studies   - Financial issues - Physical Health - Relationship between students and doctors - Bad experience during studies - Workload - Internal Pressure - External pressure - Issue in reconciling career and personal life - Work rhythm too intense - Feeling not able to | 4 (15.4)  6 (16.7)  11 (30.6)  10(27.8)  15 (41.7)  27 (75)  10 (27.8)  19 (52.8)  15 (41.7)  23 (63.9) | 0  1 (10)  5 (50)  0  3 (30)  7 (70)  3 (30)  5 (50)  3 (30)  7 (70) | 4 (15.4)  5 (19.2)  6 (23.1)  10 (38.5)  12 (46.2)  20 (76.9)  7 (26.9)  14 (53.8)  12 (46.2)  16 (61.5) | -  0.868  0243  0.058  0.615  1  1  1  0.615  9.31 |

**Supplemental Table 5** Reasons for choosing the ICM specialty and perception of the ICM speciality and comparison between beginners and fully trained residents

| Variables | All residents  N = 113 | Fully trained residents  N = 28 | Beginners  N = 85 | P value |
| --- | --- | --- | --- | --- |
| To treat life threatening emergency | 95 (84.1) | 22/78.6 | 73 (85.9) | 0.536 |
| Because leadership competence are required | 42 (37.2) | 10 (35.7) | 32 (37.6) | 1 |
| Because of the high level of technicity | 58 (51.3) | 11 (39.3) | 47 (55.3) | 0.211 |
| Because of the importance of the clinical reasoning | 104 (92) | 25 (89.3) | 79 (92.9) | 0.828 |
| Because of team work | 98 (86.7) | 22 (78.6) | 76 (84.9) | 0.252 |
| Hours are too early and late  Agree  Disagree  Neutral | 34/112 (30.4)  49/112 (43.7)  29/112 (25.9) | 15/28 (53.6)  8 (28.6)  5 (17.9) | 19 (22.6)  41 (48.8)  24 (28.6) | 0.009 |
| Too many night shifts and week ends  Agree  Disagree  Neutral | 36/110 (32.7)  34/110 (30.9)  40 (36.4) | 13/28 (46.4)  6 (21.4)  9 (32.1) | 23/82 (28)  28 (34.1)  31 (37.8) | 0.180 |
| Difficult work conditions  Agree  Disagree  Neutral | 45/112 (40.2)  40/112 (35.7)  27/112 (24.1) | 15/28 (53.6)  9 /28 (32.1)  4/28 (14.3) | 30/84 (35.7)  31/84 (36.9)  23/84 (27.4) | 0.193 |
| Utile job  Agree  Disagree  Neutral | 109/111 (98.2)  1 (0.9)  1 (0.9) | 25/27 (92.6)  1 (3.7)  1 (3.7) | 84/84 (100)  0  0 | 0.042 |
| Reconciling  personal and professional life  Agree  Disagree  Neutral | 63/110 (57.3)  18/110 (16.4)  29/110 (26.3) | 13/28 (46.4)  7/28 (25)  8/28 (28.6) | 50/82 (61)  11 (13.4)  21 (25.5) | 0.466 |

**Supplemental table 6** Comparison of the reasons for gender gap in ICM leadership position mentioned ICM residents and comparison between beginners and fully trained residents

| Variables | All residents  N = 113 | Fully trained residents  N = 28 | Beginners  N = 85 | P value |
| --- | --- | --- | --- | --- |
| Less Physical aptitude than men  Strongly disagree  disagree | 99 (87.6)  14 (12.4) | 25 (89.3)  3 (10.7) | 74 (87.1)  11 (12.9) | 1 |
| Women worried about difficulty to reconcile professional and personal life  Agree  Disagree  Neutral | 70/104 (63.4)  33/104 (30)  7/104 (6.6) | 20/28 (71.4)  7 (25)  1 (3.6) | 50/82 (61)  26/82 (31.7)  682(7.3) | 0.568 |
| System discriminates against women  Agree  Disagree  Neutral | 52/102 (51)  24 /102(23.5)  26/102 (25.5) | 16/25 (64)  8 (32)  1 (4) | 36/78 (46.2)  16/78 (20.5)  26/78 (33.3) | 0.017 |
| Lack of self confidence  Agree  Disagree  neutral | 27/109 (24.8)  59/109 54.1)  23/109 (21.1) | 7/26  10/26  9/26 (34.6) | 20/83 (24.1)  49/83 (59)  14 (16.9) | 0.102 |
| Lack of female model in ICM  Agree  Disagree  neutral | 81 (73.6)  16 (14.5)  13 (11.8) | 17/28 (60.7)  6/28 (21.4)  5 (17.9) | 64/82 (78)  10/82 (12.2)  8/82 (9.8) | 0.198 |
| Women are told to prioritise family and that is not compatible with responsibilities  Agree  Disagree  neutral | 72/110 (65.5)  29 (26.4)  9 (8.1) | 20/26 (76.9)  4/26 (15.4)  2/26 (7.7) | 52/84 (61.9)  25/84 (29.8)  7 (8.3) | 0.325 |
| Random effect  Agree  Disagree  neutral | 3 (2.9)  93/101 (92.1)  5/101 (5) | 0  22  1 (4.3) | 3/78 (3.8)  71/78 (91)  4/78 (5.1) | 0.623 |
| Masculine specialty  Agree  Disagree  neutral | 25 /105 (23.8)  59/105 (56.2)  21/105 (20) | 8/25 (32)  14/25 (56)  3/25 (12) | 17/80 (21.3)  45/80 (56.2)  18 (22.5) | 0.373 |
| Women are less encourage to get leadership position | 80 /111 (72.1)  26/111 (23.4)  5 /111 (4.5) | 21/27 (77.8)  5/27 (18.5)  1/27 (3.7) | 59/84 (70.2)  21/84 (25)  4/84 (4.8) | 0.749 |
